# The incidence and risk factors analysis of acute kidney injury in hospitalized patients received diuretics: A single-center retrospective study

**DOI:** 10.3389/fphar.2022.924173

**Published:** 2022-07-22

**Authors:** Ruiqiu Zhang, Yanxin Liu, Jia Cao, Jiahui Lao, Baobao Wang, Siwen Li, Xin Huang, Fang Tang, Xiao Li

**Affiliations:** ^1^ Department of Clinical Pharmacy, The First Affiliated Hospital of Shandong First Medical University and Shandong Provincial Qianfoshan Hospital, Shandong Engineering and Technology Research Center for Pediatric Drug Development, Shandong Medicine and Health Key Laboratory of Clinical Pharmacy, Jinan, China; ^2^ School of Basic Medicine and Clinical Pharmacy, China Pharmaceutical University, Nanjing, China; ^3^ Center for Big Data Research in Health and Medicine, The First Affiliated Hospital of Shandong First Medical University and Shandong Provincial Qianfoshan Hospital, Jinan, China; ^4^ Department of Nephrology, The First Affiliated Hospital of Shandong First Medical University and Shandong Provincial Qianfoshan Hospital, Jinan, China

**Keywords:** diuretics, acute kidney injury, pharmacoepidemiology, miss diagnosis, risk factors, logic regression model

## Abstract

Diuretics have been one of the well-known nephrotoxic drugs which can lead to acute kidney injury (AKI). However, there are few real-world studies on the incidence of AKI in hospitalized patients received diuretics. In the present study, a single-center retrospective study was conducted in our center. The clinical data of hospitalized patients received diuretics from January 2018 to December 2020 was retrospectively analyzed. Among the 18,148 hospitalized patients included in the study, 2,589 patients (14.26%) were judged as incidence with AKI, while only 252 patients were diagnosed with AKI in the medical record. Among diuretics drugs in the study, the incidence rate of AKI with torasemide was the highest with 21.62%, and hydrochlorothiazide had the lowest incidence rate (6.80%). The multiple logistic regression analysis suggested that complicated with hypertension, anemia, pneumonia, shock, sepsis, heart failure, neoplastic diseases, combined use of proton pump inhibitors (PPI) were independent risk factors for AKI related to diuretics. The logic regression models for diuretics related AKI were developed based on the included data. The model for diuretics-AKI achieved the area under the receiver operating characteristic curves (AUC) with 0.79 on 10-fold cross validation. It is urgent to improve the understanding and attention of AKI in patients received diuretics for medical workers, and the assessment of risk factors before the use of diuretics should be contributed to the early prevention, diagnosis and treatment of AKI, and ultimately reducing morbidity and improving prognosis.

## Introduction

Acute kidney injury (AKI) is a series of clinical syndromes in which serum creatinine (Scr) concentrations increase over a short period of time, or urine output decreases. It has become an increasing global concern (Lameire et al., 2013). In the past years, several studies have been conducted to address the risk of AKI in hospitalized patients due to medication uses (Susantitaphong et al., 2013; Yang et al., 2015; Fenna et al., 2019; Safadi et al., 2020). Many pathological conditions can lead to AKI, including sepsis, critical illness, circulatory shock, radiocontrast agents, and nephrotoxic drugs (Susantitaphong et al., 2013). Drug-induced acute kidney injury (D-AKI) refers to kidney injury caused by drugs or their metabolites within 7 days after the use of one or more drugs. The kidneys are rich in blood flow and have the function of acidifying the urine, making them an easy target for drug toxicity. Besides, there are enzymes in the kidney that metabolize some drugs, and if these drugs are metabolized abnormally in the kidney, substances toxic to the kidney may be produced (Safadi et al., 2020; Shi et al., 2022). It was found that about 20% of AKI in hospitalized patients was caused by medications (Pannu and Nadim, 2008; Awdishu and Mehta, 2017; de Figueiredo et al., 2017; Perazella, 2018; Liu et al., 2021; Perazella and Rosner, 2022; Zhou et al., 2022). A Chinese multicenter AKI survey showed that 71.6% of patients with AKI had a history of potentially nephrotoxic drug use before or during kidney injury (Yang et al., 2015).

Diuretics are one of the well-known nephrotoxic drugs, since they can directly or indirectly cause a significant decrease in renal blood perfusion and glomerular filtration rate through the mechanism of affecting tubulobulb feedback, which leads to kidney ischemia and hypoxia (Wu et al., 2014; Khan et al., 2017; Pierson-Marchandise et al., 2017; Liu et al., 2021; Perazella and Rosner, 2022). However, there are few real-world studies on the incidence of AKI in hospitalized patients received diuretics. In this study, we aimed to explore the incidence and risk factors analysis of AKI in hospitalized patients received diuretics and develop the logistic regression model for diuretics related AKI based on electronic medical record data. With the individual characteristics of patients, the risk of AKI can be evaluated before receiving diuretics, which may provide useful information for clinical decision making to better prevent D-AKI.

## Materials and methods

### Ethical approval

This study was approved by the Ethics Committee of First Affiliated Hospital of Shandong First Medical University (Approval No. YXLL-KY-2022-024). There is no personally identifiable information in this manuscript.

### Study design and study objects

A retrospective cohort study was conducted based on the clinical information from the electronic medical records at the First Affiliated Hospital of Shandong First Medical University. The clinical data for this study was collected from the hospital healthcare big data platform, which integrated multi-source data from hospital information system (HIS), electronic medical records (EMR), laboratory information management system (LIS), picture archiving and communication system (PACS) and nursing information system (NIS). The clinical information included sociodemographic characteristics, comorbidities, inpatient departments, diagnostics, laboratory testing, operation, hospitalization days, hospitalization costs, and concomitant nephrotoxic medication. A multidisciplinary research team, including clinical pharmacist, clinician, epidemiologist, and statistician, was developed to ensure effective implementation of the study.

The patients were included if received treatment with diuretics and discharged from the hospital between 1 January 2018 and 31 December 2020. We excluded the patients who met any of the following criteria: 1) hospital stay < 48 h; 2) Age < 18 years; 3) GFR < 30 ml/min/1.73 m^2^ within 48 h after admission; 4) AKI was diagnosed on admission; 5) less than two Scr test results during hospitalization; 6) the Scr values were always lower than 40 μmol/L during hospitalization; 7) cases with incomplete medical history information.

### Case definition

The diagnosis of AKI was based on Scr changes in accordance with the 2012 Kidney Disease: Improving Global Outcomes (KDIGO) Clinical Practice Guideline for AKI (Kellum et al., 2013). AKI can be diagnosed if one of the following criterias is met: 1) within 48 h, the absolute value of Scr increased ≥ 0.3 mg/dl (26.5 μmol/L); 2) known or speculated increase of Scr within 7 days ≥1.5 times of baseline value; 3) urine volume ≤ 0.5 ml/kg/h for more than 6 h. The correlation between drugs and AKI was mainly judged by clinical pharmacists and clinicians in the department of nephrology according to the time logic relationship. If disagreements arose during the process, they discussed them with a third party until agreement was reached.

### Statistical analysis

R software (version 3.6.3) was used for all data analyses in this study. For continuous variables, the Kolmogorov Smirnov (K-S) test was used to assess whether the data were normally distributed. The data obeying the normal distribution were expressed by mean ± SD, and the difference between groups was tested by *t* test or analysis of variance. The data disobeying the normal distribution were expressed by the median and interquartile spacing, and comparison between groups adopted Wilcoxon rank sum test. For counting data, the number of cases (n) or constituent ratio (%) were used to express. Chi-square test was employed for comparison between groups. The investigated variables were taken as independent variables and AKI groups as dependent variables, and the chi-square test was used for univariate analysis. Then the variables with *p* < 0.05 in univariate analysis were included in multivariate logistic regression analysis to identify the significant independent risk factors of acute kidney injury caused by aminoglycosides in hospitalized patients. The analysis results were expressed in odds ratio (OR) and 95% confidence interval (95% CI). All *p* values are 2-sided, and a *p* value of <0.05 was deemed significant.

### Logistic regression modeling

The logistic regression has become one of the most widely used analytical tools in the medical information field (Davis et al., 2017), due to the ability to calculate correlation coefficients between predictors and targets, and the ease of understanding and finding correlated factors. In the present study, the logistic regression models were developed with routinely collected patient information. The models were validated with 10-fold cross validation. The Receiver Operating Characteristic (ROC) and the Area Under the ROC Curve (AUC) were employed to assess the performance of the models. For highly unbalanced datasets, ROC curves and AUC are very useful and appropriate metrics.

## Results

### Incidence and clinical features of acute kidney injury patients received diuretics

There were 18,148 patients prescribed with diuretics included in the analysis. Among them, 2,589 patients could be diagnosed with AKI according to KDIGO 2012 guidelines, the incidence of diuretics related AKI was 14.26% (2,589/18,148).

In the AKI group, there were 1,643 males (63.46%) and 946 females (36.54%), and there were 9,711 males (62.41%) and 5,851 females (37.59%) in the non-AKI group, as shown in Table 1. There was no significant gender difference in the incidence of AKI (*χ* ^2^: 1.03, *p* = 0.31).

**TABLE 1:** Diuretics related AKI and patient characteristics.

Group	AKI (*n* = 2,589)	Non-AKI (*n* = 15,559)	Total (*n* = 18,148)	*χ* ^2^/Ζ	*p*-value
Gender					
Male	1,643 (63.46%)	9,711 (62.41%)	11,354 (62.56%)	1.04	0.31
Female	946 (36.54%)	5,848 (37.59%)	6,794 (37.44%)		
Age	67 (55,79)	65 (54,76)	66 (54,76)	18,767,274	<0.001
Age					
Youth	217 (8.38%)	1,402 (9.01%)	1,619 (8.92%)	21.92	<0.001
Middle age	962 (37.16%)	6,453 (41.47%)	7,415 (40.86%)		
Old age	1,410 (54.46%)	7,704 (49.52%)	9,114 (50.22%)		
Smoking					
Yes	918 (35.46%)	5,528 (35.53%)	6,446 (35.52%)	0.01	0.94
No	1,671 (64.54%)	10,031 (64.47%)	11,702 (64.48%)		
BMI (kg/m^2^)^a^	23.70 (21.23, 26.57)	23.89 (21.36, 26.56)	23.88 (21.33, 21.56)	4,575,502	0.42
BMI					
Underweight	72 (7.89%)	588 (7.25%)	660 (7.32%)	0.79	0.85
Normal	388 (42.49%)	3,474 (42.86%)	3,862 (42.83%)		
Overweight	303 (33.19%)	2,756 (34.00%)	3,059 (33.92%)		
Obesity	913 (10.02%)	1,287 (15.89%)	1,437 (15.93%)		
Hospital stay^a^	15.94 (10.06, 24.11)	15.19 (10.04, 22.79)	15.28 (10.04, 22.93)	19,509,685	<0.001
Spend^a^	92,593.2 (47,171.60, 142,342.37)	48,543.98 (23,408.99, 86,526.95)	53,148.11 (25,271.75, 94,629.78)	12,397,050	<0.001

a Two sample Wilcoxon rank sum test was used. Because some patients did not have height information during hospitalization, BMI was missing.

The median age of AKI group was 67.0 (54, 75) years, which was slightly higher than that of non-AKI group with 65.0 (50, 69) years (*p* < 0.001). The incidence rate of young group (18–40 years old), middle age group (41–65 years old) and elderly group (66 years old or above) were 13.40% (217/1,619), 12.97% (962/7,415), 15.47% (1,410/9,114) respectively, among which the elderly group had the highest incidence of AKI (*χ* ^2^: 21.92, *p* < 0.001). For smoking history, there was no difference between AKI and non-AKI groups (35.46% vs. 35.53%, *p* = 0.94). The nutritional status of the patients was divided into four groups based on Body Mass Index (BMI), including underweight (BMI < 18.5), normal weight (18.5 ≤ BMI < 24), overweight (24 ≤ BMI < 28), and obesity (BMI ≥ 28). The median BMI of AKI group was 23.70 (21.33, 26.64), the median BMI in the non-AKI group was 23.89 (21.36, 26.56), and there was no statistical significance between the two groups (*p* = 0.42).

In AKI group, the median length of stay of hospitalized patients was 15.94 (10.06, 24.11) days, and the median total cost of hospitalization was 92,593.2 (47,171.60, 142,342.37) Chinese yuan (CNY). While, the median length of stay of inpatients in non-AKI group was 15.19 (10.04, 22.79) days, and the median total cost of hospitalization was 48,543.98 (23,408.99, 86,526.95) CNY. The length of stay and total cost of patients in AKI group were significantly higher than those in non-AKI group (*p* < 0.001).

The 2,589 patients with AKI were distributed in 36 clinical departments of the hospital. As shown in Table 2, the departments with high incidence of AKI include Intensive Care Unit (ICU), Neurology, Neurosurgery, Vascular surgery, Hepatology, Cardiac surgery, Internal Medicine-Cardiovascular Department, Urology.

**TABLE 2:** Departments with high risk of diuretic related AKI.

Admission department	Number of patients using diuretics	Number of AKI cases	Incidence rate/%
ICU	2,218	641	28.90
Neurology	821	182	22.17
Neurosurgery	1,392	261	18.75
Vascular surgery	280	47	16.79
Hepatology	1,543	248	16.07
Cardiac surgery	1,069	168	15.72
Internal medicine-cardiovascular department	1937	241	12.44
Urology	1,037	100	9.64

Among the patients included in this study, the incidence of AKI was highest in those receiving torasemide at 21.62% (996/4,604). On the basic condition of hospitalization, the median length of stay and the median cost of hospitalization in AKI group were also higher than those in non-AKI group. As shown in Supplementary Table S1, the total cost of the current hospitalization in AKI and non-AKI patients with different drugs.

### Miss-diagnosis of patients with diuretic related acute kidney injury

By consulting the in-hospital medical records of adult inpatients included in this study, only 9.73% (252/2,589) were diagnosed as acute kidney injury by doctors during hospitalization. All other patients met the diagnostic criteria for AKI in terms of changes in kidney function and all indicators during their hospital stay. However, the diagnosis was not confirmed, with an underdiagnosis rate of 90.27% (2,337/2,589). The patients diagnosed as AKI on the first page of inpatient medical records are mainly distributed in departments of ICU, cardiovascular medicine, neurology, Hepatology, neurosurgery, and oncology.

### Comorbidities and concomitant therapies

In the study, the hospitalized patients with AKI usually had a variety of diseases at the same time, as shown in Table 3. The results suggested that there were significant differences for the combination of basic diseases between the two groups, including hypertension (*χ* ^2^: 31.37, *p* < 0.001), diabetes (*χ* ^2^: 14.43, *p* < 0.001), cerebral apoplexy (*χ* ^2^: 190.76, *p* < 0.001), anemia (*χ* ^2^: 103.48, *p* < 0.001), coronary heart disease (*χ* ^2^: 13.45, *p* < 0.001), pneumonia (*χ* ^2^: 450.21, *p* < 0.001), shock (*χ* ^2^: 906.74, *p* < 0.001), sepsis (*χ* ^2^: 161.90, *p* < 0.001), heart failure (*χ* ^2^: 149.16, *p* < 0.001), skin tissue infection (χ^2^: 7.66, *p* = 0.006), neoplastic diseases (*χ* ^2^: 45.93, *p* < 0.001), chronic kidney insufficiency (*χ* ^2^: 9.06, *p* = 0.003), chronic obstructive pulmonary disease (*χ* ^2^: 11.64, *p* < 0.001), hypokalemia (*χ* ^2^: 10.19, *p* < 0.001), hyponatremia (*χ* ^2^: 8.31, *p* < 0.001), liver injury (*χ* ^2^: 26.43, *p* < 0.001), acidosis (*χ* ^2^: 200.97, *p* < 0.001).

**TABLE 3:** Comorbidities and concomitant therapies.

	AKI (*n* = 2,589)	Non-AKI (*n* = 15,559)	Total (*n* = 18,148)	*χ* ^2^	*p*-value
Comorbidity					
Hypertension	1,302 (50.29%)	6,904 (44.37%)	8,206 (45.44%)	31.37	<0.001
Diabetes	728 (28.12%)	3,831 (24.62%)	4,559 (25.12%)	14.43	<0.001
Cerebral apoplexy	1,087 (41.99%)	4,434 (28.50%)	5,521 (30.42%)	190.76	<0.001
Anemia	481 (18.58%)	1781 (11.45%)	2,262 (12.46%)	103.48	<0.001
Coronary disease	988 (38.16%)	5,360 (34.45%)	6,384 (35.18%)	13.45	<0.001
Pneumonia	1,074 (41.48%)	3,428 (22.03%)	4,502 (24.80%)	450.21	<0.001
Shock	437 (16.88%)	465 (2.99%)	902 (4.97%)	906.74	<0.001
Sepsis	86 (3.32%)	97 (0.62%)	183 (1.01%)	161.90	<0.001
Heart failure	377 (14.56%)	1,147 (7.37%)	1,524 (8.40%)	149.16	<0.001
Skin tissue infection	26 (1.00%)	85 (0.55%)	111 (0.61%)	7.66	0.006
Neoplastic disease	507 (19.58%)	4,015 (25.81%)	4,522 (24.92%)	45.93	<0.001
Gout	20 (0.77%)	98 (0.63%)	118 (0.65%)	0.70	0.40
Chronic kidney insufficiency	156 (6.03%)	724 (4.65%)	880 (4.85%)	9.06	0.003
Pancreatitis	48 (1.85%)	247 (1.59%)	295 (1.63%)	0.99	0.32
Thrombocytopenia	7 (0.27%)	22 (0.14%)	29 (0.16%)	2.31	0.13
Chronic obstructive pulmonary disease	88 (3.40%)	355 (2.28%)	443 (2.44%)	11.64	<0.001
Hypokalemia	154 (5.95%)	702 (4.51%)	856 (4.72%)	10.19	<0.001
Hyponatremia	105 (4.06%)	465 (2.99%)	570 (3.14%)	8.31	<0.001
Liver injury	25 (0.97%)	45 (0.29%)	70 (0.38%)	26.43	<0.001
Liver cirrhosis	257 (9.93%)	1,608 (10.33%)	1865 (10.28%)	0.40	0.53
Hyperlipidemia	63 (2.43%)	446 (2.29%)	509 (2.80%)	1.53	0.22
Acidosis	133 (5.14%)	185 (1.19%)	318 (1.75%)	200.97	<0.001
Combination therapy					
NASID	901 (34.80%)	5,109 (32.84%)	6,010 (33.12%)	3.87	0.04
ARB	480 (18.54%)	2,631 (16.91%)	3,111 (17.14%)	4.15	0.04
ACEI	180 (6.95%)	1,304 (8.38%)	1,484 (8.18%)	6.03	0.01
PPI	419 (16.18%)	3,989 (25.64%)	4,408 (24.29%)	107.88	<0.001
Aminoglycoside	357 (13.79%)	2064 (13.27%)	2,421 (13.34%)	0.53	0.47
β-lactam drugs	603 (23.29%)	6,877 (44.20%)	7,480 (41.22%)	400.51	<0.001

As shown in Table 3, several combined medications, including nonsteroidal anti-inflammatory drugs (NASID) (*χ* ^2^: 3.87, *p* = 0.04), angiotensin IIreceptor inhibitor (ARB) drugs (*χ* ^2^: 4.15, *p* = 0.04), angiotensin converting enzyme inhibitor (ACEI) drugs (*χ* ^2^: 6.03, *p* = 0.01), proton pump inhibitor (PPI) (*χ* ^2^: 107.88, *p* < 0.001), β-Lactam drugs (*χ* ^2^: 400.51, *p* < 0.001) had significant difference between AKI and non-AKI groups.

In the AKI group, 15.99% (414/2,589) of patients underwent surgery during hospitalization, 5.26% (136/2,589) underwent cardiac surgery and 10.39% (269/2,589) underwent angiographic surgery. In the non-AKI group, 68.87% (10,715/15,559) underwent surgery, of which 4.52% (703/15,559) and 11.85% (1,843/15,559) underwent cardiac surgery and angiography, respectively.

### Analysis of risk factors in patients with diuretic related acute kidney injury

The variables with significant difference (*p* < 0.05) between AKI group and non-AKI group were selected for multivariate logistic regression to further analyze the relevant risk factors. The results are shown in Table 4, which suggested that several risk factors were related to AKI in hospitalized patients, including operation (OR: 2.39, 95% CI: 2.11∼2.71), hypertension (OR: 1.13, 95% CI: 1.01∼1.26), diabetes (OR: 0.94, 95% CI: 0.84∼1.05), cerebral apoplexy (OR: 1.56, 95% CI: 1.40∼1.74), pneumonia (OR: 1.51, 95% CI: 1.36∼1.66), shock (OR: 3.09, 95% CI: 2.624∼3.645), neoplastic disease (OR: 0.89, 95% CI: 0.79∼1.01), heart failure (OR: 1.58, 95% CI: 1.35∼1.84), chronic kidney insufficiency (OR: 0.64, 95% CI: 0.50∼0.80), acidosis (OR: 2.47, 95% CI: 1.91∼3.19), liver injury (OR: 1.32, 95% CI: 0.72∼2.34), combined use of PPI (OR: 1.41, 95% CI: 1.25∼1.60), combined use of NSAIDs (OR: 1.25, 95% CI: 1.12∼1.40), combined use of ARB (OR: 1.20, 95% CI: 1.06∼1.37), combined use β-Lactam drugs (OR: 2.06, 95% CI: 1.85∼2.29).

**TABLE 4:** Multivariate logistic regression analysis of AKI in hospitalized patients.

Variable	*β*	Wald value	OR and 95% CI	*p*-value
Age	−0.01	1.50	0.99 (0.99∼1.00)	0.22
Operation				
Operation	0.87	185.41	2.39 (2.11∼2.71)	<0.001
Contrast examination	−0.137	2.74	0.88 (0.75∼1.02)	0.10
Laboratory Values				
Scr	0.01	90.65	1.02 (1.01∼1.04)	<0.001
Leukocyte count	0.02	33.04	1.02 (1.01∼1.03)	<0.001
Platelet count	−0.01	34.93	0.99 (0.92∼1.00)	<0.001
Red blood cell count	−0.14	20.62	0.87 (0.82∼0.92)	<0.001
Uric acid	0.01	154.39	1.02 (1.01∼1.03)	<0.001
Total bilirubin	0.01	123.19	1.01 (1.01∼1.04)	<0.001
β-2 microglobulin	0.06	128.03	1.06 (1.05∼1.08)	<0.001
Comorbidity				
Hypertension	0.12	4.90	1.13 (1.01∼1.26)	0.03
Diabetes	−0.07	1.31	0.94 (0.84∼1.05)	0.25
Cerebral apoplexy	0.45	66.66	1.56 (1.40∼1.74)	<0.001
Anemia	−0.05	0.45	0.95 (0.83∼1.10)	0.50
Coronary heart disease	−0.05	0.69	0.95 (0.84∼1.07)	0.41
Pneumonia	0.41	64.37	1.51 (1.36∼1.66)	<0.001
Shock	1.13	181.56	3.09 (2.62∼3.65)	<0.001
Sepsis	0.19	1.13	1.21 (0.85∼1.73)	0.29
Skin tissue infection	0.12	0.21	1.13 (0.66∼1.88)	0.65
Neoplastic diseases	−0.12	3.67	0.89 (0.79∼1.01)	0.01
Heart failure	0.46	34.48	1.58 (1.35∼1.84)	<0.001
Chronic kidney insufficiency	−0.45	15.17	0.64 (0.50∼0.80)	<0.001
Hypokalemia	0.02	0.03	1.11 (0.82∼1.26)	0.86
Hyponatremia	0.12	0.84	1.13 (0.87∼1.45)	0.36
Liver injury	0.28	0.85	1.32 (0.72∼2.34)	0.03
Chronic Obstructive Pulmonary Disease	0.21	2.39	1.24 (0.94∼1.62)	0.12
Acidosis	0.90	47.45	2.47 (1.91∼3.19)	<0.001
Combination therapy				
NSAIDs	0.22	15.50	1.25 (1.12∼1.40)	<0.001
ACEI	0.01	0.02	1.01 (0.84∼1.22)	0.89
ARB	0.19	7.99	1.20 (1.06∼1.37)	0.01
PPI	0.35	29.81	1.41 (1.25∼1.60)	<0.001
β-lactam drugs	0.72	170.55	2.06 (1.85∼2.29)	<0.001

Torasemide was the diuretic drug with the highest incidence of AKI in this study, and furosemide as the most used diuretic in our center. We analyzed risk factors for AKI in patients using these two diuretics, respectively. As shown in Supplementary Table S2, the risk factors associated with AKI in hospitalized patients using torasemide were: operation (OR: 2.03, 95% CI: 1.66∼2.50), cerebral apoplexy (OR: 1.42, 95% CI: 1.20∼1.68), pneumonia (OR: 1.28, 95% CI: 1.08∼1.52), shock (OR: 2.24, 95% CI: 1.74∼2.88), acidosis (OR: 1.97, 95% CI: 1.29∼3.00), combined use of PPI (OR: 1.34, 95% CI: 1.09∼1.65), combined use of β-lactam drugs (OR: 1.88, 95% CI: 1.57∼2.25). For furosemide, the risk factors associated with AKI in hospitalized patients were: operation (OR: 2.82, 95% CI: 2.41∼3.31), cerebral apoplexy (OR: 1.55, 95% CI: 1.36∼1.78), pneumonia (OR: 1.68, 95% CI: 1.48∼1.93), sepsis (OR: 2.40, 95% CI: 1.51∼3.98), heart failure (OR: 1.79, 95% CI: 1.47∼2.18), neoplastic diseases (OR: 0.79, 95% CI: 0.68∼0.92), acidosis (OR: 2.13, 95% CI: 1.49∼3.03), combined use of NASIDs (OR: 1.21, 95% CI: 1.06∼1.39), combined use of β-lactam drugs (OR: 2.22, 95% CI: 1.94∼2.56), combined use of PPI (OR: 1.42, 95% CI: 1.22∼1.67), which can be seen in SupplementaryTable S3.

### Relationship between laboratory indicators and diuretic related acute kidney injury

The comparison of several important laboratory indicators between the AKI group and the non-AKI group was shown in Table 5. The indicators used were the most recent values of test results obtained prior to the use of the study drug. As shown in the table, several important indicators were significantly different between the two groups, including serum creatinine (Scr), leukocyte count, platelet count, red blood cell count, uric acid, total bilirubin, and β-2 microglobulin.

**TABLE 5:** Distribution of laboratory values across AKI and non-AKI.

	AKI (*n* = 2,589)	Non-AKI (*n* = 15,559)	χ^2^/Ζ	*p*-value
Scr	45 (0.02,967)	41 (0.01,1267)	0.54	<0.001
Leukocyte count	9.425 (0.02,184.11)	7.55 (0.01,512.62)	15,428,595.50	<0.001
Platelet count	165 (1,1123)	198 (1,1304)	23,543,825	<0.001
Red blood cell count	3.235 (0.99,6.49)	3.66 (0.79,8.44)	24,535,121.50	<0.001
Uric acid	312.9 (25.20,1123)	265 (26.20,1466)	16,813,088	<0.001
Total bilirubin	14 (1.10,727.50)	11.6 (0.40,736.20)	171,197,838.50	<0.001
β-2 microglobulin	4.01 (0.91,159.32)	2.53 (0.73,235.59)	12,079,198.50	<0.001

### Performance of the logistic regression models

**FIGURE 1:**
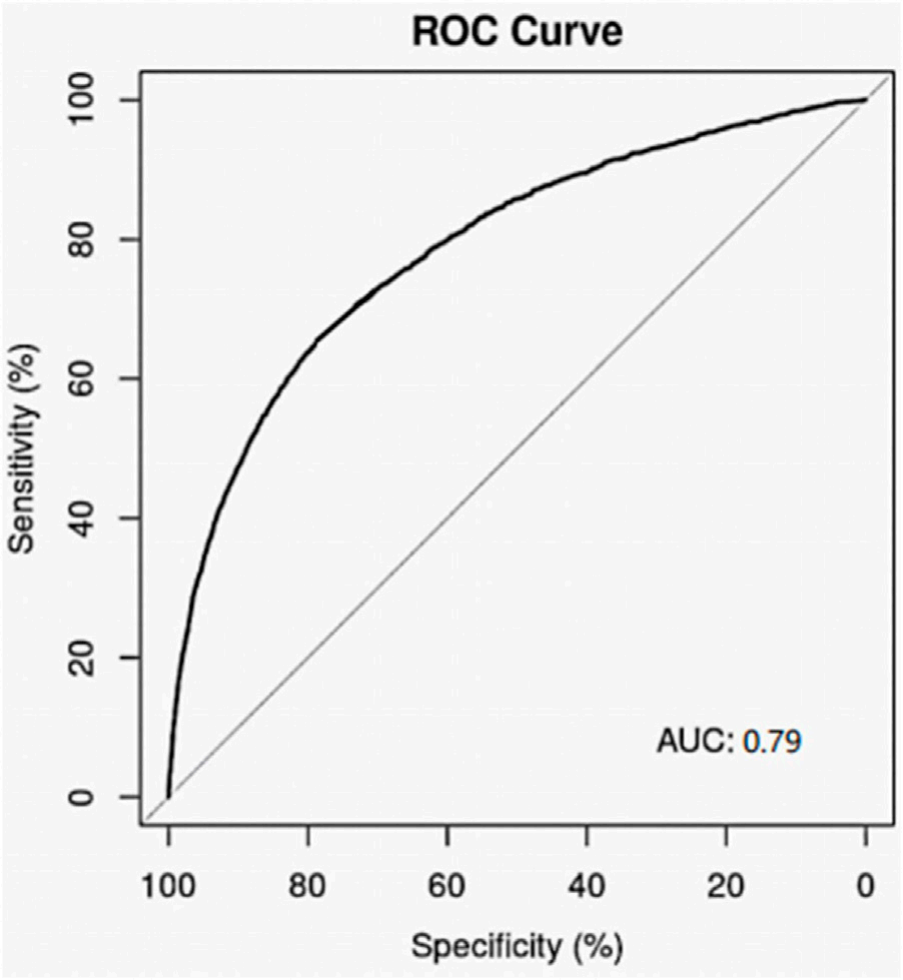
Performance of logic regression model for diuretics related AKI.

**FIGURE 2:**
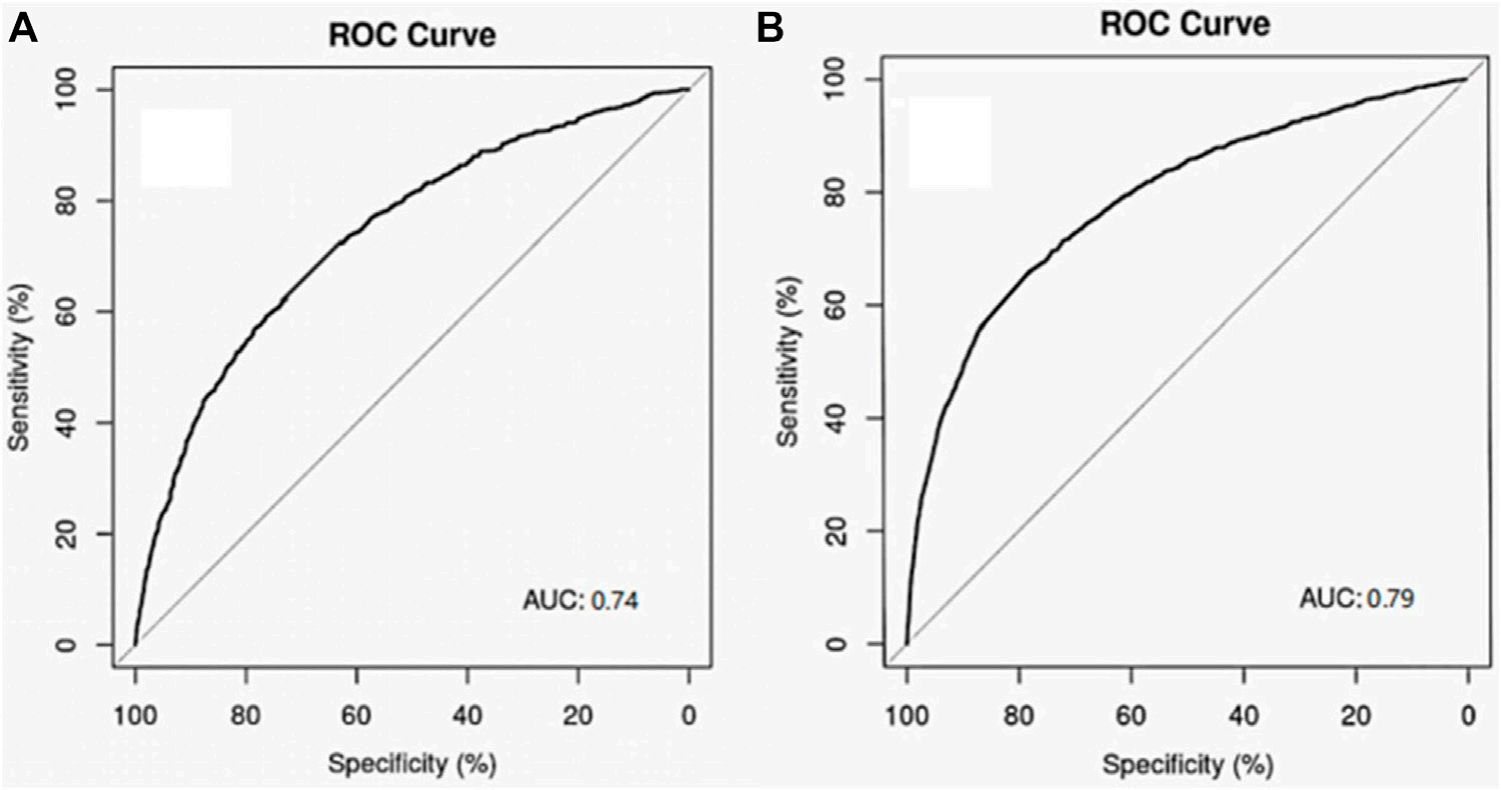
Performance of logic regression models for AKI related to torasemide and furosemide. **(A)** Torasemide; **(B)** furosemide.

The logistic regression models were built with the meaningful variables obtained from single factor analysis. The performance of the model was evaluated using the ROC curves and AUC values. For the overall patients received diuretics, the ROC curve of the model on 10-fold cross validation was shown in Figure 1. It provided the AUC value with 0.79 (95% CI: 0.78∼0.80). For torasemide with the highest incidence of AKI and furosemide with the highest utilization rate in this study, ROC curves of the model on 10-fold cross validation were shown in Figure 2. They provided the AUC values with 0.74 (95% CI: 0.72–0.0.76) and 0.79 (95% CI: 0.77–0.80), respectively.

## Discussion

### Incidence and analysis of acute kidney injury

The overall incidence rate of diuretics associated AKI with KDIGO-AKI diagnostic criteria was 14.26% (2,589/18,148) in our center. It was reported that the in-hospital AKI detection rate in China was 2.3% (Yang et al., 2015) and a study showed that the incidence rate of AKI in adult hospitalized patients was 10.7% in South Africa (Fenna et al., 2019). The diuretic drug torasemide provided the highest incidence rate of AKI (21.62%) in our center. This may be related to the large dose and long-term use of torasemide (Wu et al., 2014). The incidence of AKI in female patients was lower than male patients (13.92% vs. 14.47%), which was consistent with previous studies. This protective effect may be due to sex hormones by affecting cells in the pathogenesis of AKI (Hutchens et al., 2008; Hutchens et al., 2012). The patients in the AKI group were older than those in the non-AKI group. This may be due to the reduced physical condition of elderly patients, including renal structure and function, physiological and metabolic functions. Elderly patients often have multiple comorbidities, such as hypertension, coronary heart disease and anemia, which was related to the fact that elderly patients have a combination of multiple diseases and a greater variety of medications (Khan et al., 2017; Formica et al., 2018).

Moreover, both length of stay in hospital and total cost of hospitalization in the AKI group were higher than those in non-AKI group (*p* < 0.001). Besides, the proportion of patients with AKI admitted to ICU was higher, which increased the hospitalization expenses of patients to a certain extent. The huge expenses brought by AKI and its complications to patients should not be underestimated.

### Missed diagnosis of diuretic related acute kidney injury

Among the hospitalized patients included in the study, only 9.73% (252/2,589) cases were clearly diagnosed as AKI in medical records, the missed diagnosis rate was quite high. The high missed diagnosis rate may be due to that kidney injury and kidney function monitoring were generally lacking. Therefore, it is extremely urgent to improve kidney safety. For several departments with high diagnosis rate, the clinical indicators were paid closer attention. The changes of indicators reflecting kidney function, such as creatinine and urine volume, can be found timelier. It can result in early diagnosis of acute kidney injury and the prognosis of patients. The diagnosis of AKI mainly relied on the changes of serum creatinine and urine volume. The lack of continuous monitoring or low attention of dynamic kidney function indicators may lead to more serious missed diagnoses. Besides, the strength of inference on the causality was thus weakened given the nature of retrospective study.

The adequate dynamic monitoring of kidney indicators and enough attention of the medical team must be helpful to improve the diagnosis rate of AKI caused by diuretics. In addition, we also recommend that clinical pharmacists be provided to the department needing special attention, since the clinical pharmacists are more aware of drug-induced diseases (Viktil and Blix, 2008; Guignard et al., 2015; Gervais et al., 2021; Schulz et al., 2021). The sufficient dynamic monitoring of renal indicators and enough attention from the medical team will be beneficial to improve the diagnosis rate of AKI. It should be useful for improving the prognosis and outcome of AKI patients with early prevention and early treatment.

### Analysis of risk factors of diuretic related acute kidney injury

The surgery during hospitalization may contribute to the increased probability of AKI. It is generally believed that the operations can lead to changes in hemodynamics, activation of inflammatory mediators (Ortega-Loubon et al., 2016), and the basic pathological manifestation is acute tubular necrosis. Among the risk factors inducing AKI in patients, the combination of one or more underlying diseases or the occurrence of complications in the course of treatment are unfavorable to the occurrence and development of AKI. In this study, the injury after medication may be greater and more prone to AKI for patients who suffered from the underlying diseases, including hypertension, cerebral apoplexy, pneumonia, shock, neoplastic diseases, heart failure, chronic renal insufficiency, liver injury, acidosis. These diseases had an adverse impact on AKI patients, increasing the incidence of AKI in hospitalized patients. In addition to the influence of basic diseases, nephrotoxic drugs have always been the biggest risk factor for AKI. The results on combination medications showed that the combined use of diuretics and the other nephrotoxic drugs (PPI, ARB, NSAIDs, and β-lactam drugs) can increase the risk of kidney injury, especially for the elderly patients with more underlying diseases and more types of combined drugs. Kidney structure, function, and hemodynamics change in elderly individuals, and multiple diseases often occur simultaneously, further increasing the risk of exposure to nephrotoxic drugs. Age is therefore a risk factor for AKI (Rosner, 2013). The occurrence of AKI would lead to economic and medical burden to patients. We suggested the risk factors for AKI need to be fully assessed before the patients receive diuretics to avoid the incidence of AKI.

### Limitations of this study

There were several limitations for our study related to the retrospective design. First, in the present study, AKI was diagnosed based on the dynamic changes in Scr independently of urine output, which would have missed some AKI cases. Second, the determination of AKI was based on reviewing medical records and therefore might result in misdiagnosis in some cases. Third, the diagnosis of AKI emphasizes the change of creatinine in a short time, but the monitoring of creatinine was not adequate in our center. Lastly, the patients with missed clinical test indicators and inaccurate test results were eliminated in the study, this may bring bias on the final statistical results to a certain extent.

## Conclusion

The investigation indicated the high incidence rate of diuretics associated AKI (14.26%) in our center, while more than 90% cases were not detected and diagnosed in time. It is urgent to improve the understanding and attention of AKI for medical workers. Several risk factors for diuretics associated AKI were screened out, including old age, admission to ICU, suffering underlying diseases (hypertension, stroke, anemia, pneumonia, shock, and heart failure), combined use of PPI and β-Lactam drugs. Based on the meaningful variables obtained from single factor analysis, we developed the logistic regression models for AKI risk in hospitalized patients received diuretics. The model for diuretics-AKI performed well and the AUC value was 0.79 on 10-fold cross validation. Before using diuretics, full evaluation of the risk factors and close attention to the indexes of kidney function must be helpful to the decrease of diuretic related AKI. Meanwhile, it is necessary to improve the cognition and incidence rate of AKI among medical staff and patients. We suggested the incidence of AKI in hospitalized patients received diuretics should be taken seriously, and the risk factors for AKI need to be fully assessed before the patients receive diuretics to avoid the incidence of AKI.

## Data Availability

The original contributions presented in the study are included in the article/Supplementary Material, further inquiries can be directed to the corresponding author.
