# Supplementary material for: The incidence and risk factors analysis of acute kidney injury in hospitalized patients received diuretics: A single-center retrospective study
Source: Front Pharmacol. 2022 Jul 22;13:924173. doi: 10.3389/fphar.2022.924173 (PMC9355122; doi:10.3389/fphar.2022.924173)
Supplement: Supplementary file 1 [file DataSheet1.docx]

**Table S1** Clinical features of AKI patients received torasemide

|  | **AKI(n=996)** | **Non AKI**  **(n=3610)** | **χ^2^/Ζ** | ***P* Value** |
| --- | --- | --- | --- | --- |
| **Gender** | | | | |
| Male | 650(65.26%) | 2367(65.57%) | 0.032 | 0.857 |
| Female | 346(34.74%) | 1243(34.43%) |  |  |
| **Age** | | | | |
| Youth | 69(6.93%) | 292(8.09%) | 3.722 | 0.155 |
| Middle age | 356(35.74%) | 1365(37.81%) |  |  |
| Old age | 571(57.33%) | 1953(54.10%) |  |  |
| **Smoking** | | | | |
| Yes | 378(37.95%) | 1349(37.37%) | 0.113 | 0.736 |
| No | 618(62.05%) | 2261(62.63%) |  |  |
| Hospital stay* | 15.025(2.01,168.11) | 14.955(2.02,160.66) | 1858217 | 0.104 |
| Spend* | 62919.805(380.36,903498.49) | 56467.825(3122.28,618788) | 1236988 | <0.001 |

**Two sample Wilcoxon rank sum test was used.*

**Table S2** Multivariate logistic regression analysis of torasemide related AKI

| **Variable** | **β** | **Wald Value** | ***OR* and 95%*CI*** | ***P* Value** |
| --- | --- | --- | --- | --- |
| **Operation** | | | | |
| Operation | 0.707 | 45.783 | 2.029(1.657~2.497) | <0.001 |
| **Laboratory Values** | | | | |
| Scr | 0.002 | 22.822 | 1.002(1.001~1.003) | <0.001 |
| Leukocyte count | 0.008 | 6.032 | 1.009(1.002~1.016) | 0.014 |
| Platelet count | -0.001 | 6.980 | 0.999(0.998~0.999) | 0.008 |
| Red blood cell count | -0.025 | 0.271 | 0.974(0.885~1.072) | 0.602 |
| Uric acid | 0.001 | 28.190 | 1.001(1.000~1.001) | <0.001 |
| Total bilirubin | 0.004 | 42.691 | 1.004(1.002~1.005) | <0.001 |
| β- 2 microglobulin | 0.064 | 53.599 | 1.067(1.049~1.086) | <0.001 |
| **Comorbidity** | | | | |
| Hypertension | 0.081 | 0.927 | 1.084(0.919~1.278) | 0.336 |
| Cerebral apoplexy | 0.351 | 16.601 | 1.420(1.199~1.682) | <0.001 |
| Anemia | -0.016 | 0.020 | 0.984(0.788~1.223) | 0.866 |
| Pneumonia | 0.245 | 7.855 | 1.278(1.076~1.516) | 0.005 |
| Shock | 0.807 | 39.157 | 2.241(1.739~2.884) | <0.001 |
| Sepsis | 0.028 | 0.012 | 1.209(0.615~1.706) | 0.911 |
| Heart failure | 0.233 | 3.826 | 1.262(0.997~1.592) | 0.050 |
| Skin tissue infection | 0.287 | 0.588 | 1.333(0.626~2.744) | 0.443 |
| Acidosis | 0.677 | 9.913 | 1.968(1.288~2.998) | 0.002 |
| **Combination therapy** | | | | |
| ACEI | -0.009 | 0.003 | 0.990(0.720~1.344) | 0.953 |
| β-Lactam drugs | 0.629 | 47.964 | 1.877(1.572~2.246) | <0.001 |
| PPI | 0.294 | 7.838 | 1.342(1.094~1.653) | 0.005 |

**Table S3** Multivariate logistic regression analysis of furosemide related AKI

| **Variable** | **β** | **Wald Value** | ***OR* and 95%*CI*** | ***P* Value** |
| --- | --- | --- | --- | --- |
| **Operation** | | | | |
| Operation | 1.035 | 165.186 | 2.817(2.409~3.305) | <0.001 |
| **Laboratory Values** | | | | |
| Scr | 0.003 | 63.819 | 1.003(1.002~1.0040) | <0.001 |
| Leukocyte count | 0.023 | 36.712 | 1.024(1.016~1.032) | <0.001 |
| Platelet count | -0.001 | 30.835 | 0.998(0.997~0.998) | <0.001 |
| Red blood cell count | -0.181 | 22.580 | 0.834(0.773~0.898) | <0.001 |
| Uric acid | 0.002 | 117.813 | 1.002(1.001~1.002) | <0.001 |
| Total bilirubin | 0.003 | 77.389 | 1.003(1.002~1.004) | <0.001 |
| β- 2 microglobulin | 0.049 | 56.079 | 1.050(1.037~1.064) | <0.001 |
| **Comorbidity** | | | | |
| Hypertension | 0.115 | 2.804 | 1.122(0.980~1.284) | 0.094 |
| Diabetes | -0.034 | 0.226 | 0.966(0.838~1.112) | 0.634 |
| Cerebral apoplexy | 0.441 | 40.986 | 1.554(1.357~1.778) | <0.001 |
| Anemia | -0.047 | 0.271 | 0.953(0.795~1.139) | 0.602 |
| Coronary heart disease | -0.073 | 0.981 | 0.930(0.805~1.072) | 0.322 |
| Pneumonia | 0.526 | 60.110 | 1.682(1.481~1.932) | <0.001 |
| Sepsis | 0.875 | 13.971 | 2.400(1.512~3.798) | <0.001 |
| Heart failure | 0.583 | 34.376 | 1.792(1.472~2.175) | <0.001 |
| Neoplastic diseases | -0.231 | 9.404 | 0.793(0.683~0.918) | 0.002 |
| Chronic obstructive pulmonary disease | 0.141 | 0.582 | 1.151(0.792~1.639_ | 0.445 |
| Hypokalemia | 0.068 | 0.244 | 1.071(0.811~1.399) | 0.621 |
| Hyponatremia | 0.103 | 0.375 | 1.109(0.789~1.535) | 0.540 |
| Liver injury | 0.772 | 3.585 | 2.165(0.959~4.784) | 0.058 |
| Acidosis | 0.756 | 17.472 | 2.131(1.489~3.030) | <0.001 |
| **Combination therapy** | | | | |
| NASIDs | 0.194 | 7.508 | 1.214(1.056~1.394) | 0.006 |
| ARB | 0.134 | 2.645 | 1.143(0.971~1.342) | 0.104 |
| β-Lactam drugs | 0.799 | 129.498 | 2.224(1.940~2.555) | <0.001 |
| PPI | 0.351 | 19.214 | 1.421(1.216~1.666) | <0.001 |
